# Combined spinal cord stimulation and intrathecal Morphine–Sufentanil therapy for refractory cancer pain: a case report

**DOI:** 10.3389/fragi.2026.1710432

**Published:** 2026-01-28

**Authors:** Zhi Shan Zhang, Yi Zhang, Jie Wu

**Affiliations:** 1 Department of Pain Management, Chang Zhi People’s Hospital Affiliated to Chang Zhi Medical College, Chang Zhi, Shanxi, China; 2 Department of Pain Management, China-Japan Friendship Hospital Affiliated to the Chinese Academy of Medical Sciences, Beijing, China

**Keywords:** intrathecal drug delivery System, Morphine, refractory cancer Pain, spinal cord stimulation, Sufentani

## Abstract

This case report describes the management of a 61-year-old female patient with metastatic adenoid cystic carcinoma and chronic refractory cancer pain. Initially treated with spinal cord stimulation (SCS), she later developed tolerance and inadequate analgesia. An intrathecal drug delivery system (IDDS) was subsequently implanted, and combination therapy with intrathecal morphine and sufentanil was initiated. This regimen achieved significant pain relief, reduced Numerical Rating Scale (NRS) scores, and improved sleep quality as measured by the Pittsburgh Sleep Quality Index (PSQI). The case highlights the potential efficacy of combining neuromodulation and intrathecal analgesia in managing complex cancer pain. However, due to limited data on sufentanil use in IDDS and the absence of standardized conversion protocols, further studies are warranted.

## 1 Introduction

Global malignant tumor incidence continues to rise. Concurrent advances in modern oncology have substantially prolonged patient survival (Snijders et al., 2023). Consequently, cancer-related pain has become the predominant symptom in oncology patients, rendering refractory pain management a critical medical and societal challenge (Wang and Jin, 2017). Neuromodulation techniques—particularly spinal cord stimulation (SCS)—have gained prominence in managing intractable cancer pain (Fan et al., 2021). Complementing these approaches, intrathecal drug delivery systems (IDDS)—pioneered by Wang et al., 's 1979 subarachnoid morphine application—remain globally accepted for chronic cancer pain (Liu and Yang, 2019). Nevertheless, the complex pathogenesis of refractory cancer pain necessitates ongoing therapeutic refinement. Hence, identifying optimal novel drug combinations is a key research priority (Wang and Jin, 2017). In this context, we implemented combined intrathecal morphine and sufentanil therapy in a refractory cancer pain patient, achieving clinically meaningful pain relief. Elderly cancer patients frequently display altered pharmacokinetics and heightened susceptibility to opioid-related cognitive impairment and falls. The present case, characterised by severe cachexia (BMI 15.6 kg/m^2^) and long disease duration, illustrates how sequential neuromodulation and intrathecal opioid rotation may reduce systemic opioid exposure in frail, older adults.

## 2 Case description

### 2.1 General information

A 61-year-old woman (160 cm; 40 kg; BMI 15.6 kg/m^2^) was admitted in February 2025 with severe cancer cachexia. She reported a 7-year history of refractory pain with daily breakthrough episodes. Two decades earlier, adenoid cystic carcinoma (ACC) of the hard palate had been treated by radical resection; no adjuvant therapy was given. A 2017 health screening revealed a left upper-lobe nodule; subsequent video-assisted thoracoscopic wedge resection and pleural biopsy confirmed metastatic ACC. Immunohistochemistry and morphology indicated that the lung and pleural nodules represented metastases from the original hard-palate ACC. Pain emerged immediately after surgery, exhibiting both neuropathic (tearing, stabbing) and nociceptive features along T3-T6 dermatomes and the left scapular region. Paroxysms occurred 4–6 times daily; breakthrough pain intensity peaked at 9 on the 11-point NRS, with a baseline of 4–10. Despite escalating multimodal therapy—tramadol 400 mg/day, five fentanyl 4.2 mg patches, and sustained-release morphine 240 mg/day—pain control remained inadequate. Concurrently, she developed severe sleep disturbance (PSQI 15–18) and an 18% weight loss (Zhang, 2001). SCS implanted in November 2021 provided transient relief, but opioids remained necessary. Pain recurred within 1 year, prompting dose escalation. In 2024 an intrathecal drug delivery system (IDDS) was inserted; an intrathecal morphine test dose of 0.4 mg yielded 50% relief for 3 h. The 0.4 mg intrathecal morphine test dose corresponded to ≈1/60 of the preceding oral morphine equivalent (240 mg/day) and produced 50% pain relief without respiratory depression or pruritus, fulfilling our ≥2-point NRS reduction criterion for pump implantation. Subsequently, disease progression produced IDDS tolerance; despite escalation to 23 mg/day (0.575 mg/kg/day) analgesia remained incomplete.

#### 2.1.1 Physical examination on admission

Physical examination showed no spinal scoliosis. Cervical and lumbar spine range of motion was normal. Tenderness was noted in the left thoracodorsal region. Chest wall expansion was symmetric during respiration. Paraspinal tenderness and percussion pain were elicited during dorsal examination. Pain assessment showed a baseline NRS score of 4 at rest, escalating to 9 during breakthrough episodes.

#### 2.1.2 Examinations

Admission coagulation profiles and complete blood counts were unremarkable. PET-CT (February 2025) revealed left-sided pleural thickening, chest-wall infiltration, and osteoblastic lesions in the spine and ribs, together with hepatic hypermetabolic foci, indicating widespread ACC dissemination. These findings and clinical history indicate malignant recurrence with multisystem metastatic involvement.

#### 2.1.3 Diagnoses

1. Chronic intractable cancer pain of mixed nociceptive–neuropathic type (NRS 9/10); 2. ACC with metastases to lung, pleura, bone, and liver; 3. severe cancer cachexia (BMI 15.6 kg/m^2^); 4. intrathecal opioid tolerance.

## 3 Treatment and follow-up

A diagnosis of primary lung malignancy was confirmed on July 4, 2017, leading to video-assisted thoracoscopic wedge resection of the left lung mass with concurrent pleural biopsy under general anesthesia. Postoperatively, the patient developed persistent left thoracodorsal pain. Gabapentin was titrated from 300 mg/day to 900 mg/day over 4 weeks; at 900 mg/day her NRS remained 5-6 and DN4 score 5–10, leading us to judge inadequate neuropathic pain control. A further increase to 1,800 mg/day was precluded by dizziness and somnolence. Thus, we considered the gabapentin trial sufficient but ineffective, prompting neuromodulation.

On 3 November 2021 she was admitted for refractory pain (NRS 9; PSQI 17–20) that had escalated despite oral tramadol. A trial of intrathecal hydromorphone was undertaken before planned IDDS implantation. Pain decreased from 9 to 4 for ≈2 h, but intolerable constipation and emesis precluded permanent pump insertion. DN4 questionnaire score was 5, indicating cancer-related neuropathic pain with poor morphine sensitivity (Truini et al., 2023). Given the indolent nature of her metastatic ACC (with a 20-year history) and the neuropathic component of her pain (as evidenced by the DN4 score), which is less responsive to morphine, spinal cord stimulation (SCS) was evaluated as the next therapeutic option.

The SCS lead was implanted on 8 November 2021. Gabapentin was stopped postoperatively and tramadol given intermittently, reducing pain to NRS 3 and PSQI 6–9. By Nov 2022 pain recurred (NRS 3–5) and SCS efficacy was deemed lost. Disease progression led to SCS tolerance, rib metastases with osteolysis, and a shift toward predominant nociceptive pain.

One year after SCS implantation, pain control deteriorated. This was accompanied by disease progression, including rib metastases with osteolysis, suggesting a shift towards a more predominant nociceptive pain component. Given that IDDS is particularly suited for managing diffuse or mechanical cancer-related pain, and the patient had developed tolerance to systemic and intrathecal opioids, we decided to explant the SCS and proceed with IDDS implantation. IDDS implantation with SCS explantation was performed on February 5, 2024. Under fluoroscopic guidance, catheter placement via L2-3 paramedian approach positioned the tip at the inferior endplate of T7. Intrathecal morphine (10 mg/mL) was initiated at 3 mg/day via pump, achieving significant analgesia (NRS 3, PSQI 6) and enabling systemic opioid tapered from 240 mg/day oral morphine equivalent to 30 mg/day within 4 weeks.

**FIGURE 1:**
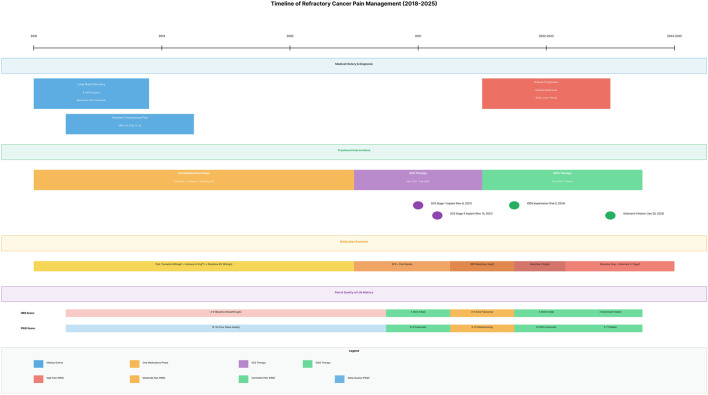
SCS implant → loss of efficacy → IDDS implant → opioid rotation to morphine + sufentanil.

On 20 January 2025, intrathecal morphine was escalated to 23 mg/day for worsening pain, yet analgesia remained inadequate. During admission the pump was refilled with morphine HCl 200 mg plus sufentanil HCl 200 μg in 40 mL sterile water, and oral pregabalin 225 mg twice daily was added. The initial infusion rate was set at sufentanil 5 μg/day plus morphine 5 mg/day. The initial ratio of intrathecal morphine 5 mg/day to sufentanil 5 µg/day (≈1000:1) was extrapolated from the published equianalgesic data indicating that sufentanil is ∼1000-fold more potent than morphine, tempered by its faster redistribution and shorter duration after intrathecal delivery (Ummenhofer et al., 2000). Within 2 weeks pain stabilised at NRS 3 and PSQI 6, breakthrough episodes declined, sleep improved, and no adverse events were recorded. Over the subsequent 6 months the infusion was titrated to sufentanil 10 μg/day plus morphine 5 mg/day, maintaining NRS 3-4 and PSQI 5-7 without significant adverse effects (Figure 1).

## 4 Discussion

In 2018, the International Classification of Diseases (ICD) classified chronic cancer pain into subtypes including visceral, bone, neuropathic, and other types, based on the International Association for the Study of Pain’s framework (Wang and Jin, 2017; Jeff et al., 2010). SCS delivers electrical impulses via epidural electrodes to modulate spinal cord pathways, thereby inhibiting pain signal transmission. Clinical studies have established the safety and efficacy of SCS for chronic pain management (Didier et al., 2017). Over the past decade, SCS application for chronic refractory and neuropathic pain (NP) has expanded in China, with efficacy supported by domestic clinical reports and guideline recognition (Fan et al., 2022). Since 2012, implantable IDDS have been utilized for refractory cancer-related and non-cancer pain. Recent research emphasizes IDDS applications for non-cancer pain and infusion parameter optimization (Zhang et al., 2018).

### 4.1 Regarding the choice between SCS and IDDS

PACC guidelines recommend that the choice between intrathecal drug delivery (TDD) and spinal cord stimulation (SCS) be dictated by pain aetiology. For non-malignant pain, both modalities are first-line; however, SCS is preferred when the syndrome is localised and non-diffuse, whereas TDD is favoured for diffuse or mechanical cancer-related pain. In patients with end-stage malignancy, SCS can be offered as an adjunct when pain is predominantly neuropathic, tumour kinetics are indolent, and intrathecal morphine trial is inadequate (Polyanalgesic Consensus Conference PACC®, 2024).

In contrast, IDDS modifies drug delivery pathways, enabling dose reduction and adverse effect minimization, thus expanding its applicability to diverse refractory pain syndromes (Brogan et al., 2023). IDDS therapy permits individualized drug and dose selection according to pain etiology and severity (Lindsay et al., 2020). During late-stage SCS therapy, the patient exhibited inadequate pain control, escalating opioid requirements, and significant adverse effects. Consequently, IDDS implantation was initiated, providing a management paradigm for refractory cancer pain. But, long-term IDDS therapy carries risks of medication-related adverse events and opioid tolerance (The Polyanalgesic Consensus Conference PACC, 2017; Upadhyay and Mallick, 2012; Sparlin and Leon-Casasola, 2011).

### 4.2 On the combined use of sufentanil and morphine

Morphine, a classic opioid agonist, provides potent and sustained analgesia. Its mechanism of action involves diffusion into the cerebrospinal fluid, followed by binding to μ-opioid receptors in the substantia gelatinosa of the dorsal horn. This interaction modulates nociceptive transmission by inhibiting synaptic input from C-fibers and Aδ-fibers to dorsal horn neurons, thereby attenuating pain signaling (Dong, 2011). However, its clinical utility is often limited by dose-dependent adverse effects—notably nausea, pruritus, and respiratory depression—which occur even at therapeutic doses (Zhang et al., 2004; Xu et al., 2004).

Sufentanil, a fentanyl derivative and potent μ-opioid agonist, has approximately 1000-fold greater analgesic potency than morphine (see Table 1). Its high lipophilicity facilitates rapid penetration of the blood-brain barrier and binding to receptors in the spinal cord dorsal horn, thereby producing analgesia (Dong, 2011). At therapeutic doses, sufentanil provides effective spinal analgesia while inducing minimal respiratory depression and cardiovascular effects. This lipophilicity also promotes its distribution into white matter structures after intrathecal administration. However, rapid redistribution from the intrathecal space to the systemic circulation results in a relatively short duration of analgesic effect. Nevertheless, it achieves potent analgesia within 2 h after administration (Geng et al., 2006; Zhang et al., 2004; Xu et al., 2004). We selected sufentanil over ziconotide, clonidine, or bupivacaine for three reasons: first, ziconotide was unavailable in our institution and requires rigorous monitoring for cognitive side effects in elderly patients; second, in a cachectic patient (BMI 15.6 kg/m^2^) already receiving high-dose systemic opioids, clonidine and bupivacaine would have increased the risk of hypotension and orthostatic intolerance; third, sufentanil’s lipophilicity enables rapid suppression of breakthrough pain while permitting dose reduction of morphine—a pharmacological profile that aligned well with the patient’s predominant pattern of nociceptive flares. Therefore, this case presents a low-cost and readily applicable alternative for frail older adults when ziconotide is unavailable and sympatholytic adjuvants are poorly tolerated—a scenario not previously detailed in the intrathecal cancer-pain literature.

**TABLE 1:** (PACC 2017; Ummenhofer 2000).

Published intrathecal opioid equivalence in humans	
Morphine	1 mg
Hydromorphone	0.2 mg
Fentanyl	0.025 mg
Sufentanil	0.001 mg

Ratios are approximate and derived from acute pain models.

The patient exhibited a suboptimal response to high-dose intrathecal morphine (23 mg/day) and experienced significant treatment-emergent adverse effects. Further escalation of the morphine dose failed to provide adequate analgesia. The combination of morphine and sufentanil significantly reduced Numerical Rating Scale (NRS) scores and Pittsburgh Sleep Quality Index (PSQI) values, without notable adverse events such as nausea or respiratory depression. This multimodal approach consequently improved the patient’s quality of life. The efficacy likely reflects a pharmacodynamic synergy: the sustained analgesia from morphine complements the rapid-onset effect of sufentanil, thereby overcoming limitations inherent to monotherapy. This strategy reduces the morphine requirement while mitigating its dose-dependent adverse effects. The 1000:1 starting ratio was intentionally selected to counterbalance sufentanil’s rapid redistribution and to minimize morphine-related side effects, although prospective dose-finding trials are still lacking (Ummenhofer et al., 2000).

Reports on the use of sufentanil in implantable intrathecal drug delivery systems (IDDS) are limited and predominantly focus on postoperative acute pain management. Standardized protocols for combined intrathecal sufentanil-morphine therapy lack validation in large-scale clinical studies. The Polyanalgesic Consensus Conference (PACC) guidelines recommend a conversion ratio of 4:1 when switching from intrathecal morphine to fentanyl (The Polyanalgesic Consensus Conference PACC, 2017). Sufentanil is 7–10 times more potent than fentanyl and approximately 1000-fold more potent than morphine. However, reported analgesic potency ratios for intrathecal administration vary considerably across studies (Giorgio et al., 2003; Ummenhofer et al., 2000; Daewhan et al., 2019). Although the 1000:1 morphine:sufentanil ratio appeared effective in this patient, we recognise that this equivalence is extrapolated from epidural labour-analgesia studies (Ummenhofer 2000) and has not been validated in chronic intrathecal cancer-pain trials. This discrepancy in conversion ratios reflects a lack of sufficient clinical data on intrathecal analgesic combinations. The differing pharmacokinetic properties of hydrophilic and lipophilic opioids further complicate the determination of intrathecal conversion ratios. Additional clinical data are needed to establish safe and effective protocols for intrathecal therapy.

We acknowledge the concerns raised by the U.S. Food and Drug Administration (FDA) and the Centers for Disease Control and Prevention (CDC) regarding the off-label use of compounded intrathecal admixtures, as they may pose risks of physicochemical incompatibility, infection, or catheter occlusion (Polyanalgesic Consensus Conference PACC®, 2024). In the present case, both morphine and sufentanil were commercially available, preservative-free formulations. The admixture was prepared immediately prior to pump refill under a vertical laminar flow hood, in strict compliance with our hospital pharmacy’s sterile compounding standards. To assess physical compatibility, we conducted a 2-h *in-vitro* visual inspection of the mixture. This inspection revealed no precipitation, color change, or turbidity. Furthermore, a follow-up computed tomography (CT) scan at 6 months showed no evidence of catheter obstruction or intrathecal granuloma formation. While these findings suggest short-term compatibility in this specific instance, we fully acknowledge that this admixture constitutes off-label use and lacks formal stability data. Larger prospective studies are warranted to establish the long-term safety and stability of such combinations.

### 4.3 Rationale for the sequential therapeutic approach

This case illustrates a sequential, step-up approach for managing refractory cancer pain. The initial choice of SCS was guided by the patient’s neuropathic pain characteristics and the indolent course of her disease, making her a suitable candidate for a neuromodulation device with a potentially long-term benefit. When the pain pattern evolved with disease progression, becoming more diffuse and nociceptive in nature, and coupled with SCS tolerance, the therapy was escalated to IDDS. This approach aligns with the PACC evidence hierarchy, which suggests SCS for localized neuropathic pain and IDDS for diffuse cancer-related pain or cases with opioid tolerance (Polyanalgesic Consensus Conference PACC®, 2024). Finally, the introduction of intrathecal sufentanil to morphine was a response to the development of tolerance to high-dose intrathecal morphine monotherapy, representing an opioid rotation and combination strategy within the IDDS framework.

This 61-year-old patient with a BMI of 15.6 kg/m^2^ was classified as “frail.” The sequential neuromodulation-intrathecal opioid rotation strategy avoided the gastrointestinal and central nervous system side effects associated with daily high-dose oral opioids, suggesting its potential value in similar elderly cachectic populations. Implantable devices (SCS/IDDS) carry a slightly higher risk of surgical and postoperative infection in frail elderly patients with low BMI, necessitating a comprehensive benefit-risk assessment.

## 5 Conclusion

This case demonstrates the successful use of a sequential, multimodal approach—combining spinal cord stimulation, intrathecal drug delivery, and opioid rotation with morphine and sufentanil—for managing refractory cancer pain. This case therefore illustrates a practical, low-cost escalation option when ziconotide is inaccessible and sympatholytic adjuvants are poorly tolerated in frail, elderly cancer patients. While the results are promising, the lack of standardized conversion ratios and limited clinical data on intrathecal sufentanil highlight the need for further research. Future studies should focus on optimizing drug combinations, refining patient selection criteria, and establishing evidence-based guidelines for neuromodulation and intrathecal therapies in cancer pain management.

## Data Availability

The original contributions presented in the study are included in the article/supplementary material, further inquiries can be directed to the corresponding author.
